# Multiple‐batch spawning as a bet‐hedging strategy in highly stochastic environments: An exploratory analysis of Atlantic cod

**DOI:** 10.1111/eva.13251

**Published:** 2021-06-10

**Authors:** Sara Hočevar, Jeffrey A. Hutchings, Anna Kuparinen

**Affiliations:** ^1^ Department of Biological and Environmental Science University of Jyväskylä Jyväskylä Finland; ^2^ Department of Biology Dalhousie University Halifax NS Canada; ^3^ Institute of Marine Research Flødevigen Marine Research Station His Norway; ^4^ Department of Natural Sciences University of Agder Kristiansand Norway

**Keywords:** Atlantic cod, bet‐hedging, environmental stochasticity, fitness, multiple‐batch spawning, risk‐spreading

## Abstract

Stochastic environments shape life‐history traits and can promote selection for risk‐spreading strategies, such as bet‐hedging. Although the strategy has often been hypothesized to exist for various species, empirical tests providing firm evidence have been rare, mainly due to the challenge in tracking fitness across generations. Here, we take a ‘proof of principle’ approach to explore whether the reproductive strategy of multiple‐batch spawning constitutes a bet‐hedging. We used Atlantic cod (*Gadus morhua*) as the study species and parameterized an eco‐evolutionary model, using empirical data on size‐related reproductive and survival traits. To evaluate the fitness benefits of multiple‐batch spawning (within a single breeding period), the mechanistic model separately simulated multiple‐batch and single‐batch spawning populations under temporally varying environments. We followed the arithmetic and geometric mean fitness associated with both strategies and quantified the mean changes in fitness under several environmental stochasticity levels. We found that, by spreading the environmental risk among batches, multiple‐batch spawning increases fitness under fluctuating environmental conditions. The multiple‐batch spawning trait is, thus, advantageous and acts as a bet‐hedging strategy when the environment is exceptionally unpredictable. Our research identifies an analytically flexible, stochastic, life‐history modelling approach to explore the fitness consequences of a risk‐spreading strategy and elucidates the importance of evolutionary applications to life‐history diversity.

## 1 INTRODUCTION

Natural environmental conditions change continuously across multiple spatial and temporal scales. While some of the fluctuations can be predictable and of low magnitude, others are uncertain, occurring with varying degrees of intensity, periodicity and stochasticity. Stochasticity can be integral to the shaping of genotypes, phenotypes and populations, either concomitantly or not (for a detailed review, see Lenormand et al., 2009). Environmental stochasticity influences the ecological processes of a population, determines the rate and direction of its evolutionary change (Frank & Slatkin, 1990; May, 1973) and can even lead to its extinction (Lande, 1993). Hence, to mitigate challenges arising from prevailing environmental uncertainty, organisms have evolved a diversity of life‐history strategies (Kussell & Leibler, 2005; Maynard Smith, 2010; Meyers & Bull, 2002; Moran, 1992; Stearns, 1976; Tufto, 2015). One of these is bet‐hedging (Gillespie, 1973, 1974, 1975; Slatkin, 1974).

Bet‐hedging is a costly genotypic strategy that maximizes long‐run or geometric mean fitness across generations by trading off the arithmetic mean in reproductive output and its variance (Cohen, 1966; Gillespie, 1974; Lewontin & Cohen, 1969; Seger & Brockmann, 1987; Simons, 2002; Yoshimura & Clark, 1991). In other words, bet‐hedging can act as a ‘portfolio effect’ (Markowitz, 1952) through which the diversification of assets, here partitioning of offspring among batches, reduces the risk and stabilizes the returns, that is geometric mean fitness of a genotype.

Organisms can spread risk among their offspring on a temporal or spatial scale, in a conservative or diversified way, or even as a complex combination of all the above (Haaland et al., 2019; Scheiner, 2014). While conservative bet‐hedging maximizes fitness by reducing the variance in fitness at the individual level, diversifying bet‐hedging does so by reducing the correlation in expected fitness among individuals in the same population (Starrfelt & Kokko, 2012). Examples of bet‐hedging strategies appear in a wide range of systems and forms (Childs et al., 2010; Philippi & Seger, 1989) such as iteroparity (Cole, 1954; Ranta et al., 2002), seed dormancy (Cohen, 1966; Simons, 2009), seed dispersal (Beckman et al., 2018; Snyder, 2011), flowering schedule (Simons & Johnston, 2003), timing of sexual reproduction (Tarazona et al., 2017), embryonic diapause (Furness et al., 2015) and hatching asynchrony (Laaksonen, 2004).

Simons (2011) extensively reviewed over 100 studies on bet‐hedging, categorizing them based on the strength of the empirical evidence. Although bet‐hedging life histories have been reported for a variety of species and hypothesized for even more, from bacteria (Beaumont et al., 2009) to vertebrates (Lips, 2001; Mahony & Thumm, 2002), the strength of the evidence for most has been limited or, as Simons (2011) put it, the evidence has been elusive. He proposed six, ranked evidence conditions that need to be met: (I) recognize a bet‐hedging trait; (II) monitor the unpredictable environment; (III) observe differences in the trait among populations; (IV) demonstrate differences in fitness dynamics; (V) validate whether the trait is favoured under relevant varying environments; and (VI) test the optimality of the trait under a range of conditions of fluctuating selection (Simons, 2011).

Few studies possess sufficient empirical support to fulfil the highest three and most data‐demanding categories of evidence for bet‐hedging (i.e. categories IV–VI; Simons, 2011). The majority of those that do fulfil these conditions are on plants (Childs et al., 2010; Simons & Johnston, 2003). This general lack of evidence can be attributed to the very considerable challenges of recognizing the adaptive significance of a trait that is bet‐hedged and the difficulty of tracking across‐generational fitness in a stochastically fluctuating environment. In our study, we attempt to overcome these challenges and aspire to provide support for or against the fifth evidence category on bet‐hedging significance of multiple‐batch spawning strategy.

Multiple‐batch spawning is a reproductive strategy common among marine fishes, such as gadoids and flounders, for example haddock (*Melanogrammus aeglefinus*), pollock (*Pollachius virens*), whiting (*Merlangus merlangus*), halibut (*Hippoglossus hippoglossus*) and dab (*Limanda limanda*) (Murua & Sabrido‐Rey, 2003). Yet, the fitness benefits of the multiple‐batch strategy have not been comprehensively explored. To sustain population resilience in a stochastic environment, bet‐hedging could be crucial for multiple‐batch spawning fish populations. A strategy of broadcast spawning on multiple spawning grounds, multiple times (Kjesbu, 1989) and over prolonged periods (Hutchings & Myers, 1994; Kjesbu et al., 1996) might act as a portfolio effect by reducing the risk of complete reproductive failure. The production of multiple egg batches within a spawning season, the number of which increases with female weight and body size (Kjesbu et al., 1996; Roney et al., 2018), could enable a batch spawner to spread the environmental risk among its offspring and mitigate the fitness consequences of environmental fluctuations. As a trade‐off in diversification, the variance in reproductive output of a multiple‐batch spawner could be lower, boosting the across‐generational geometric mean fitness, at the expense of producing a lower average number of offspring.

To tackle the question of whether multiple‐batch spawning yields the predicted fitness benefits of a bet‐hedging strategy, we used Atlantic cod, *G. morhua* (Linnaeus, 1758), as a focal species in this study. Atlantic cod, one of the most studied batch spawning fish species, has been speculated to be a conservative bet‐hedger (e.g. Hutchings & Rangeley, 2011), but never in fact tested for it. Here, we test this hypothesis by expanding an eco‐evolutionary model parameterized for cod (Kuparinen et al., 2012). Our primary objectives are to (i) observe how multiple‐batch spawning affects populational dynamics; (ii) evaluate the fitness consequences of multiple‐batch spawning within a spawning season, under different levels of environmental stochasticity; (iii) inspect the variance in reproductive output within generations; and (iv) analyse the proportion of successful spawning seasons.

## 2 MATERIALS AND METHODS

There can be several risk distribution strategies acting on different stages or processes in a species at any given time. This complication has potential to obscure the fitness consequences of any one component of the bet‐hedging strategy (Simons, 2011). Thus, we focused solely on the component of multiple‐batch spawning.

### 2.1 Multiple‐batch spawning and environmental stochasticity

We examined whether multiple‐batch spawning constitutes a bet‐hedging strategy by exploring its eco‐evolutionary impacts on fitness dynamics under varying levels of environmental stochasticity affecting batch survival. We did so by implementing an individual‐based mechanistic model developed by Kuparinen et al. (2012) which characterizes the eco‐evolutionary dynamics and demographic processes of Atlantic cod. The main evolving trait of the model is body size, which fits our research design since batch production, fecundity and spawning duration are size‐related traits. Given that the model's configuration and parameterization have been thoroughly described elsewhere (Kuparinen et al., 2012, 2014), we outline below only the main features (Table 1). Here, we focus on a detailed description of newly implemented batch spawning strategies and components of generated environmental stochasticity in batch survival.

**TABLE 1:** Summarizing the main underlying empirically derived variables of the eco‐evolutionary model

Variable	Description	Equation	Value	Unit	Source
*L*	Length calculated each year for every individual following the Von Bertalanffy growth curve	$L_{\left(t\right)}=L_{\infty }‐\left(L_{\infty }‐L_{0}\right)\cdot e^{‐kt}$ $\log\left(k\right)=‐0.609‐0.013\cdot L_{\infty }$	$L_{\left(t\right)}$	cm	von Bertalanffy (1938) Kuparinen et al. (2012)
*W*	Weight calculated each year for every individual from the length‐weight relationship	$W_{t\;}=3.52\cdot 10^{‐6}\cdot L_{t}^{3.19}$	$W_{\left(t\right)\;}$	kg	Kuparinen et al. (2012)
*L*_mat_	Length at maturity	$L_{\text{mat}}=0.66\cdot L_{\infty }$	*L*_mat_	cm	Jensen (1997)
*N*_batches_	Number of spawned batches calculated each year for every mature female	$N_{\text{batches}}=\frac{21.156}{1+\exp\left(\frac{55.014‐L_{\text{fork}\left(t\right)}}{10.141}\right)}$	$N_{\text{batches}\left(t\right)}$		Roney et al. (2018)
*N*_eggs_	Number of spawned eggs, calculated each year for every mature female	$N_{\mathrm{eggs}}=\;\left(\frac{0.48\cdot \left(W_{t}+0.37\right)}{1.45}+0.12\right)\cdot 10^{‐6}\;$	$N_{\text{eggs}\left(t\right)}$		Hutchings (2005)
Costs_MBS_	Costs of multiple‐batch spawning strategy, calculated each year for every mature female spawning more than one batch	${\text{Costs}}_{\text{MBS}}=\;\sum_{\text{batch}=1}^{N_{\text{batches}}}1‐0.00523\;\cdot \left(\text{batch}‐1\right)$	${\text{Costs}}_{\text{MBS}\left(t\right)}$		Roney et al. (2018)

**FIGURE 1:**
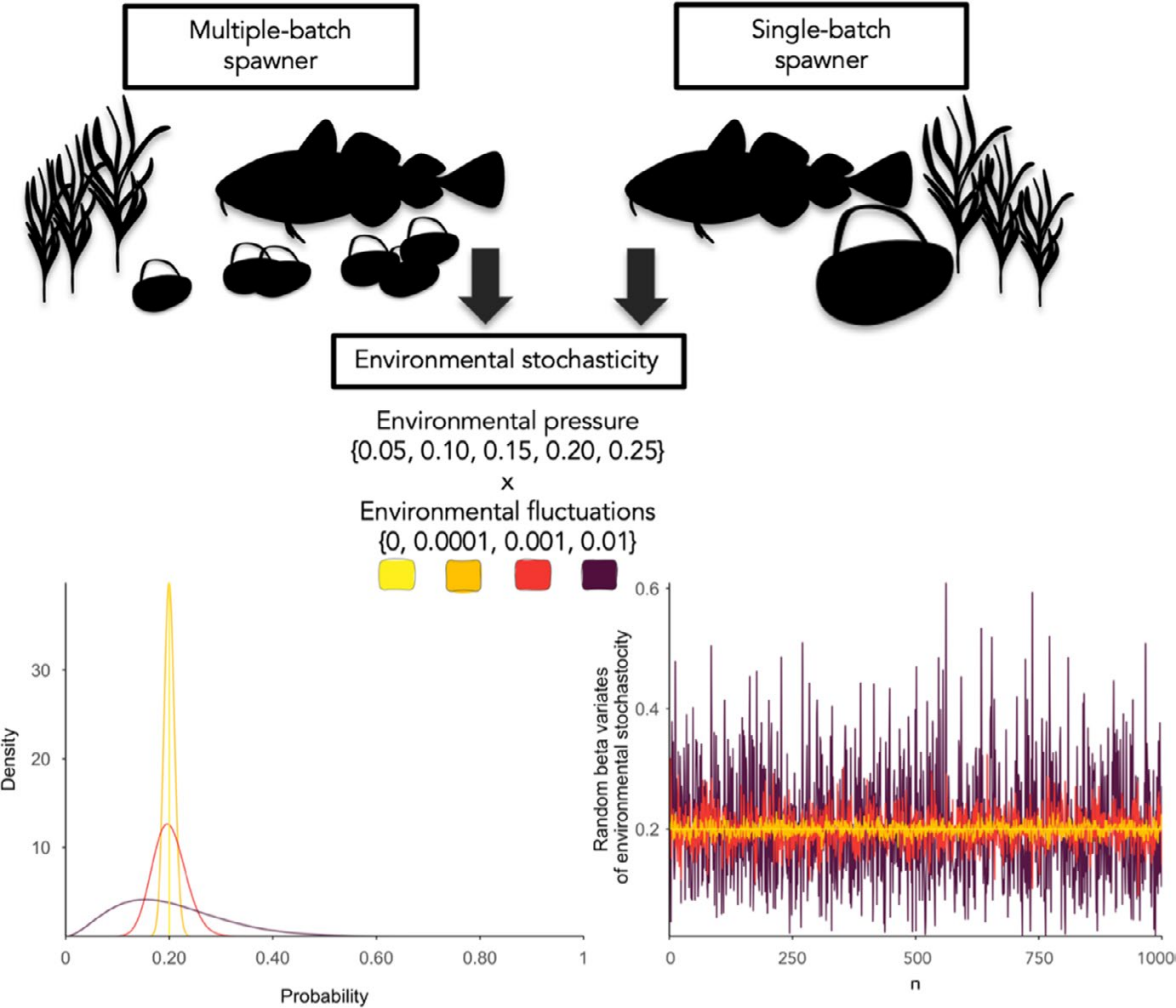
The schematic diagram demonstrates the multiple‐batch and single‐batch spawning cod populations simulated under a stochastic environment with varying rate of environmental pressure (0.05–0.25) and environmental fluctuations (0–0.01). The first graph is illustrating the probability density function for the beta distribution where mean environmental pressure applied to batch survival equals 0.20 and fluctuates depending on the environmental fluctuation rate (0, 0.0001, 0.001, 0.01). Correspondingly, the second graph is demonstrating the random, beta‐generated batch mortality rates, drawn from each probability density function

We simulated the fecundity of every mature female at the start of each spawning season through juvenile production and survival. The production of eggs was a positively dependent function of the female's weight derived from the empirically based length (*L*)–weight (*W*) relationship $W_{t\;}=3.52\cdot 10^{‐6}\cdot L_{t}^{3.19}$. The egg number was calculated based on the empirical relationship as $\text{eggs}=\left(\frac{0.48\cdot \left(W_{t}+0.37\right)}{1.45}+0.12\right)\cdot 10^{‐6}$ (Hutchings, 2005; Kuparinen et al., 2012). We portrayed the life‐history strategy of multiple‐batch spawning cod in the model as the occurrence of multiple reproductive events within a single spawning season. To highlight how the strategy feeds back on the population dynamics of cod, we compared it to a separately simulated hypothetical population with no such risk‐spreading strategy, that is single‐batch spawners. While individuals in a multiple‐batch spawning population distribute their eggs among several batches, individuals in a single‐batch spawning population only have one reproductive event within each spawning season and, thus, practise the tactic of ‘placing all your eggs in one basket’ (Figure 1). The number of eggs that a multiple‐batch spawning female produced in one spawning season was distributed evenly among batches.

**FIGURE 2:**
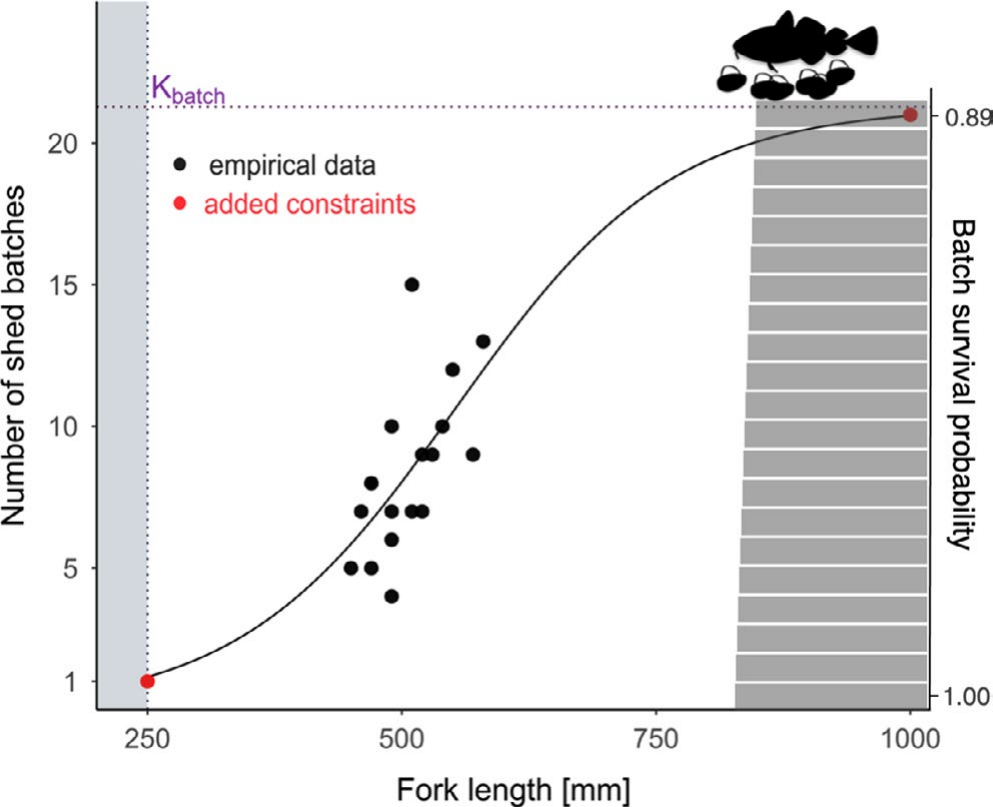
Relationship between mature female size, batch number and batch survival probability. The empirically gathered data (black) on the abundance of batches were plotted against the fork length of mature female cod and added constraints (red). A sigmoid curve given by a solid line was fitted to the data set and used in the simulation process. Vertical blue‐shaded area indicates the female size smaller than 250 mm, which were not considered mature in our model. Grey‐shaded bar chart on the right side of the figure illustrates the batch survival probability assigned to every batch according to the order at which female has shed it in the spawning period (values based on Roney et al., 2018). As a result, the batch survival probability is decreasing with increasing order number at which batch is shed (*y*‐axis)

The number of batches depended on body size. At each annual step, the number of batches for each mature female was derived from an empirically based nonlinear regression fit between the fork length $L_{\text{fork}\left(t\right)}$ and batch number $N_{\text{batches}}$, where $N_{\text{batches}}=\frac{21.156}{1+\exp\left(\frac{55.014‐L_{\text{fork}\left(t\right)}}{10.141}\right)}$ (parameterized based on an empirical data set collected from Risør fjord in coastal Skagerrak by Roney et al., 2018; Figure 2). Before the nonlinear regression was fitted, the lower value of 0 batches at size 25 cm and higher value of 21 batches at 100 cm were added to the experimentally gathered data to account for somatic constraints. We based these constraints on data of maximum observed batch number in captive Norwegian coastal cod (Kjesbu et al., 1996) and limited the function by setting the maximum available number of batches to 21 to prevent the continuous increase of produced batches with a female's size.

To make multiple‐batch spawning a costly trait, we introduced costs to batch survival (right‐sided bar plot on Figure 2). We set these costs based on empirical findings, following the spawning dynamics of 73 wild‐caught Norwegian coastal cod in Skagerrak and their offspring quality (Roney et al., 2018). Larval length and yolk‐sac volume of offspring spawned in an experimental spawning basin at the Institute of Marine Research Flødevigen exhibited declining trends during the spawning period. Given that later spawned batches produced smaller larvae at hatch, we added an assumption that shedding of the first or single batch had no associated costs, while for every consecutive batch, shed within the same season, the batch spawning costs increased. Because larvae length tends to correlate with survival probability, we applied the trade‐off in costs of batch production and risk‐spreading potential in a gradually decreasing survival probability of each batch from 1.00 to 0.89 for the first to 21st produced batch, respectively (Roney et al., 2018; following the mortality function described by Pepin, 1991). Therefore, while single‐batch spawners experienced no such batch spawning costs, as they could shed only one batch per spawning season, the average costs of multiple‐batch spawners varied depending on how many batches an individual has shed in a spawning season.

We introduced environmental stochasticity to batch survival as the environmental pressure (0.05, 0.10, 0.15, 0.20, 0.25) and environmental fluctuations (0, 0.0001, 0.001, 0.01) and varied them in a full factorial manner (Figure 1). We define environmental pressure as a change in the mean batch mortality rate that could be driven, for example by any combination of ecological, environmental or anthropogenic factors. On the other hand, environmental fluctuations are defined as a change in the variance of batch mortality rate and can be generated by the stochasticity about the ecological, environmental or anthropogenic factors. Hence, we separately exposed multiple‐batch and single‐batch spawning populations to each of 20 simulated environmental scenarios and compared their fitness performances. Higher rates of environmental pressure and fluctuations were dismissed upon trial testing, since the stochasticity became overwhelming and drove the populations simulated in our study towards extinction. When the added environmental fluctuations were preset to zero, the scenario was considered nonstochastic. Under these circumstances, we applied a constant environmental pressure to a batch or batches as a success probability in a Bernoulli trial to determine the survival of an entire batch or group of batches. While the environmental pressure under such nonstochastic scenarios remained constant, the outcome of successful survival of a batch or batches could still vary among seasons and individuals as it was newly drawn in every spawning season for every mature female.

By contrast, the stochastic scenario was characterized by the presence of a variance in the form of continuous environmental fluctuations around mean rates of environmental pressure (Figure 1). The final environmental stochastic rate applied to batches was preadjusted, based on the rate of environmental pressure and environmental fluctuation, using a beta distribution parameterized by the *α* and *β* shape parameters (see Tables S1 and S2), and was drawn for every batch in every spawning season. To derive the final survival outcome of each batch, we applied the final environmental stochastic rate as a success probability in a Bernoulli trial to each batch separately, meaning that, for each batch, we drew a random number (0 or 1) as to whether the batch either dies or survives (i.e. a predator or environmental disaster destroyed a whole batch). Subsequently, to determine the final number of offspring, we summed the number of eggs from survived batches for every multiple‐batch spawning female and multiplied the sum with a natural survival rate from the egg stage to 3‐year‐old recruit estimated to be $1.13\cdot 10^{‐6}$ for northern cod (Hutchings, 2005). The same process was adopted for the single‐batch spawning population.

Thus, we created a combination of 40 different scenarios, comprising multiple‐batch spawning populations under 20 distinct conditions of environmental stochasticity and single‐batch spawning populations under 20 distinct conditions of environmental stochasticity (Figure 1).

### 2.2 Mechanistic model of Atlantic cod

The mechanistic model (Kuparinen et al., 2012) follows the life stages of each individual fish in a population at annual time steps and combines genetic and optimization approaches through the use of heritable growth trajectories. The trajectories were derived from least square fits of empirically gathered 258 cod growth trajectories (Kuparinen et al., 2012), using the von Bertalanffy growth model $L_{\left(t\right)}=L_{\infty }‐\left(L_{\infty }‐L_{0}\right)\cdot e^{‐kt}$, where $L_{\left(t\right)}$ is the length of a fish at age t, $L_{\infty }$ is the asymptotic body length, $\;L_{0}$ is the length at t=0 and k (year − 1) is the growth coefficient which describes the rate at which $L_{\infty }$ is reached (von Bertalanffy, 1938). Two observed associations underpin the model: (i) the observed negative correlation between $L_{\infty }$ and k, where $\log\left(k\right)=‐0.609‐0.013\cdot L_{\infty }$, and (ii) the ratio of the length at maturity $L_{\text{mat}}$ and $L_{\infty }$, where $L_{\text{mat}}=0.66\cdot L_{\infty }$ (Jensen, 1997) when $30\;\text{cm}\leq L_{\infty }\leq 120\;\text{cm}$ (Kuparinen et al., 2012).

Each individual carried a genotype of 10 unlinked, diploid loci with 2 alleles (0 and 1) that followed classical Mendelian inheritance. The sum of these 10 loci, that could range from 0 to 20, coded for the genotypic value of $L_{\infty }$ and, thus, allowed for evolution of growth to occur. Ten loci were sufficient in describing the trait distribution smoothly; adding additional loci did not affect the simulations. Final phenotypic value, generated as an environmental variation (s.d. = 3.5) around the genotypic trait value, coded for the phenotypic $L_{\infty }$ value that provided a basis for the estimation of other relevant size‐based traits. To initiate the external fertilization process, a mature male was randomly assigned to a mature female, and the sex of offspring was determined by a 50/50 Bernoulli trial (Kuparinen et al., 2012).

In addition to the demographic processes of reproduction and survival described in the batch spawning component of the model, density‐dependent growth and natural mortality were also simulated at each annual time step on the individual level, and the state of the population was tracked accordingly. Growth of each individual was defined by its von Bertalanffy parameters ($L_{\infty }$ and k) and additionally altered by density‐dependent population dynamics. Time available for growth at each annual step was bounded between 0 and 1. If the population reached or exceeded the carrying capacity, the individual's growth time and progress along its growth trajectory was reduced in accordance with a logistic equation $e^{15‐17.6\cdot c}{\left(1+e^{15‐17.6\cdot c}\right)}^{‐1}$, where c is the ratio between the biomass of the population and its carrying capacity. Populations of multiple‐batch and single‐batch spawners had the same preset carrying capacity in all scenarios. At low population density, the individual's time spent on growth within one year was close to 1, allowing an individual almost a full annual growth increment along its von Bertalanffy curve (see Figure S1). Therefore, the population density affected fecundity by regulating the individual's growth, which impacts (i) the time the individual needs to reach 66% of its asymptotic length and mature and (ii) the age when reproduction starts.

An instantaneous rate of natural mortality rate of 0.15, which was not applied until individuals had reached 3 years of age (see above), was assumed to be equal for all individuals of age 3 years or older (Kuparinen et al., 2012). If the individual was mature, the mortality rate was additionally increased by 0.10 to account for the survival cost of reproduction (following Kuparinen et al., 2012), resulting in an instantaneous rate of 0.25, which corresponds with the estimated natural mortality of many cod populations (Beverton et al., 1994). Using the binomial distribution, the model simulated the survival of every individual at each annual step with the maximum lifespan set to 25 years.

### 2.3 Simulation design

To achieve reproducibility of the code and results, we initialized a pseudorandom number generating sequence in a repeatable manner before each scenario run in R software (R Core Team, 2019) and allowed the loop to iterate and produce 50 replica simulations. As bet‐hedging is predicted to have the greatest benefits in stochastic environments (Simons, 2011), we produced several combinations of runs to generate quantitative data (Figure 1). In the run of each scenario, we initialized the simulations with a preadapted cod population to separately simulate a population consisting of only multiple‐batch spawners and a population consisting of only single‐batch spawners under each of the environmentally stochastic scenarios for 5000 years. This time interval was sufficient as it was beyond the time needed for the populations to reach their dynamic eco‐evolutionary equilibriums (Figure S2).

To investigate the potential underlying feedbacks of the bet‐hedging strategy on life histories, we looked into the dynamics of the last 2500 years of the population in each run by recording the population variables and life‐history traits at each annual step. The primary recorded output data were total number of recruits produced in one year, and annual population averages of $L_{\infty }$ and k, abundance, biomass and mortality rate. These outputs of 50 replica simulations per run were then summarized across replicates by recording the mean, coefficient of variance and standard deviation of each variable in every year.

For the last 300 years of each simulation process, we tracked the individual fitness of every mature fish and recorded its total number of successfully shed batches, batch size and realized reproductive output over the individual's lifespan. The total reproductive output of every mature individual was recorded as the sum of the realized number of 3‐year‐old recruits produced in the lifetime of the original individual. To explore whether multiple‐batch spawning is a bet‐hedging strategy of Atlantic cod, we looked into across‐generational fitness elements of multiple‐batch spawning populations under simulated environmentally stochastic scenarios and compared them to a single‐batch spawning population. Across‐generational fitness elements included the arithmetic mean fitness ${\bar{W}}_{\text{AM}}$, variance in arithmetic mean fitness among generations or cohorts, and geometric mean fitness ${\bar{W}}_{\text{GM}}$. We measured the arithmetic mean fitness ${\bar{W}}_{\text{AM}}$ and its variance across generations for each scenario as an average realized lifetime reproductive output of a generation and the across‐generational geometric mean in fitness ${\bar{W}}_{\text{GM}}$ as the nth root of the product of average realized lifetime reproductive output of every generation following ${\bar{W}}_{\text{GM}}={\left({\bar{W}}_{\text{AM}1}\cdot {\bar{W}}_{\text{AM}2}\cdot \ldots \cdot {\bar{W}}_{\text{AM}n}\right)}^{1/n}$ (Seger & Brockmann, 1987), where n is a number of generations or cohorts.

Fitness outputs were pooled together per run, and the mean, variance and coefficient of variation of each variable were recorded. Statistical analyses of relationships and trends were done using Welch's two‐sample *t* test (Welch, 1947), nonparametric Kruskal–Wallis test (Kruskal & Wallis, 1952) and simple linear regression (Kenney & Keeping, 1962). All simulations and analyses were performed in the open‐source software R (R Core Team, 2019), and figures were produced using the package tidyverse (Wickham et al., 2019).

## 3 RESULTS

### 3.1 Population dynamics of each of the spawning strategists

Separately simulated multiple‐batch and single‐batch spawning cod populations exposed to 20 variations of environmentally stochastic scenarios (Figure 1) were analysed to investigate whether the costly spawning of multiple batches might be considered a bet‐hedging strategy in Atlantic cod. In all 2000 simulations (2 spawning strategies * 20 different environmental scenarios * 50 replica simulations of each spawning population), the populations adapted from initial conditions to the new specific environments in fewer than 2500 years, maintaining a stable dynamic thereafter (Figure S2).

Mean population size for all scenarios was 5311 individuals (s.d. 623 individuals across scenarios) and exhibited a declining trend (up to a 33.10% decrease) as the environmental fluctuations and pressure on batch survival or batch mortality rate increased. Coefficient of variation in population size was greater overall for single‐batch spawning populations (Table S3) and significantly increased with increasing environmental pressure on batch survival: from 2.28 (CV) under the least pressured environmental scenarios to 3.20 (CV) under the most pressured environmental scenarios in multiple‐batch spawning populations, and from 2.37 (CV) under the least pressured environmental scenarios to 3.52 (CV) under the most pressured environmental scenarios in single‐batch spawning populations.

Environmental fluctuations had no significant effect on either population size or CV in population size of single‐batch spawners but did affect multiple‐batch spawners (Table S4), causing a significant difference between scenarios with and without any applied environmental fluctuations. In particular, population size was more variable under scenarios with no environmental fluctuations as it varied from s.d. 165 individuals to s.d. 134 individuals under scenarios with environmental fluctuations. However, there was no significant variation among scenarios with different environmental fluctuation rates. The same dynamics were observed for population biomass and its CV (Tables S3 and S4).

Multiple‐batch spawning cod populations had significantly lower realized average mortality rate compared to single‐batch spawning populations (Figure S3a). While the realized mortality rate did not differ among different environmental fluctuating rates in single‐batch spawning populations, it increased significantly in multiple‐batch spawning populations when environmental fluctuations were applied to each batch separately (Table S4).

### 3.2 Fitness components

**FIGURE 3:**
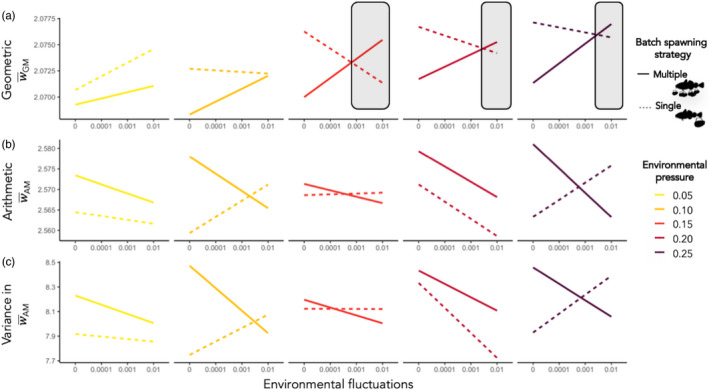
Trends of three fitness components: across‐generational geometric mean in fitness (${\bar{W}}_{\text{GM}}$), across‐generational average reproductive output or arithmetic mean in fitness (${\bar{W}}_{\text{AM}}$) and across‐generational variance in arithmetic mean fitness (variance in ${\bar{W}}_{\text{AM}}$) plotted against increasing rate of environmental fluctuations (*x*‐axis), and grouped by increasing environmental pressure applied to batch survival (colours). Linear regression trends, that were based on the average value of each observed variable across all generations per every run of each spawning type, are illustrated by a solid line for multiple‐batch spawners and by a dashed line for single‐batch spawners. Grey‐shaded area indicates the environmental conditions under which a costly multiple‐batch spawning evolutionary outperforms the single‐batch spawning strategy

**FIGURE 4:**
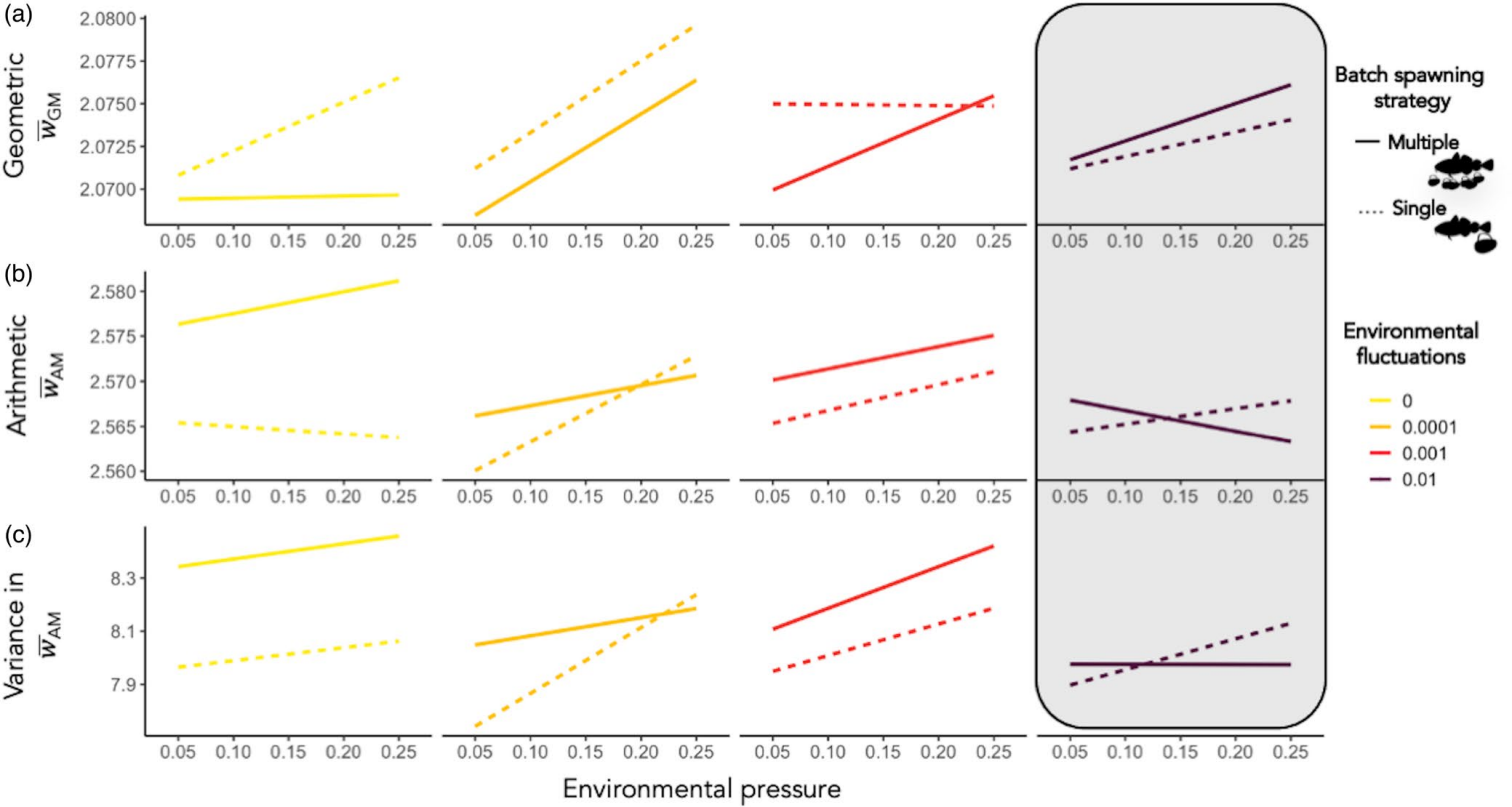
Trends of three fitness components: across‐generational geometric mean in fitness (${\bar{W}}_{\text{GM}}$), across‐generational average reproductive output or arithmetic mean in fitness (${\bar{W}}_{\text{AM}}$) and across‐generational variance in arithmetic mean fitness (variance in ${\bar{W}}_{\text{AM}}$) plotted against increasing rate of environmental pressure (*x*‐axis), and grouped by increasing environmental fluctuations applied to batch survival (colours). Linear regression trends, that were based on the average value of each observed variable across all generations per every run of each spawning type, are illustrated by a solid line for multiple‐batch spawners and by a dashed line for single‐batch spawners. Grey‐shaded area indicates the environmental conditions under which multiple‐batch spawning strategy constitutes a bet‐hedging, as the ${\bar{W}}_{\text{GM}}$ increases at the cost of reduced ${\bar{W}}_{\text{AM}}$

Under increasing environmental fluctuations, multiple‐batch spawners experienced a significant increasing trend in long‐run geometric mean fitness ${\bar{W}}_{\text{GM}}$, resulting in a higher long‐run ${\bar{W}}_{\text{GM}}$ under most unpredictable and uncertain environmental conditions (Figure 3a). Both strategies had an increasing trend in ${\bar{W}}_{\text{GM}}$ with increasing environmental pressure (Figure 4a), but the relationship was significant, albeit weak, only in multiple‐batch spawning populations (Table S6). The difference in ${\bar{W}}_{\text{GM}}$ across all environmentally stochastic scenarios differed significantly between multiple‐batch and single‐batch spawning populations by being lower in the multiple‐batch spawning population (Table S5) as their ${\bar{W}}_{\text{GM}}$ values were lower under conditions when the environmental fluctuations were absent or low, and/or the environmental pressure was weak.

The variance in ${\bar{W}}_{\text{AM}}$ across‐generations was higher overall in multiple‐batch spawning populations (Table S5) and had a significant decreasing trend with increasing environmental fluctuations (Figure 3c; Table S6). This significance and decreasing trend were due to higher variance in ${\bar{W}}_{\text{AM}}$ among generations under nonstochastic conditions, while the variance among single‐batch spawning cod generations increased in the presence of environmental fluctuations. In contrast, environmental pressure had no significant effect on the variance in ${\bar{W}}_{\text{AM}}$ among generations of either multiple‐ or single‐batch spawners.

Multiple‐batch spawners had overall significantly higher arithmetic mean in fitness ${\bar{W}}_{\text{AM}}$ compared to single‐batch spawners (Table S5) due to greater realized reproductive output in the absence of environmental fluctuations (Figure 3b). While the environmental pressure had no significant effect on ${\bar{W}}_{\text{AM}}$ for either strategy, the presence of environmental fluctuations significantly decreased the ${\bar{W}}_{\text{AM}}$ of multiple‐batch spawners (Table S6). This effect resulted in a lower ${\bar{W}}_{\text{AM}}$ for multiple‐batch spawners when exposed to the highest environmental pressure and environmental fluctuations, hence, experiencing elevated environmental uncertainty and mortality (Figure 3b and Figure 4b).

The environmental scenarios where the three fitness components: (i) high long‐run geometric mean fitness ${\bar{W}}_{\text{GM}}$, (ii) low arithmetic mean in fitness ${\bar{W}}_{\text{AM}}$ and (iii) low across‐generational variance in ${\bar{W}}_{\text{AM}}$ overlap illustrate that multiple‐batch spawning is a bet‐hedging strategy (Figure 4 grey‐shaded area).

### 3.3 Variance in reproductive output within generations

Multiple‐batch spawning populations had a lower variance in within‐generational reproductive output than single‐batch spawning populations (Figure S3 and Table S3). The two components of environmental stochasticity—the environmental fluctuations and environmental pressure applied to batch mortality—had a significant effect on the variance in within‐generational reproductive output of multiple‐batch spawning populations (Table S4). However, post hoc Dunn's test revealed that the effects were significant only due to the increased variance under the most pressured environments (0.25) and predictable environmental conditions (0). The scenarios with environmental fluctuations (0.0001, 0.001, 0.01) did not differ significantly in the within‐generational variance of reproductive output.

### 3.4 Spawning success

**FIGURE 5:**
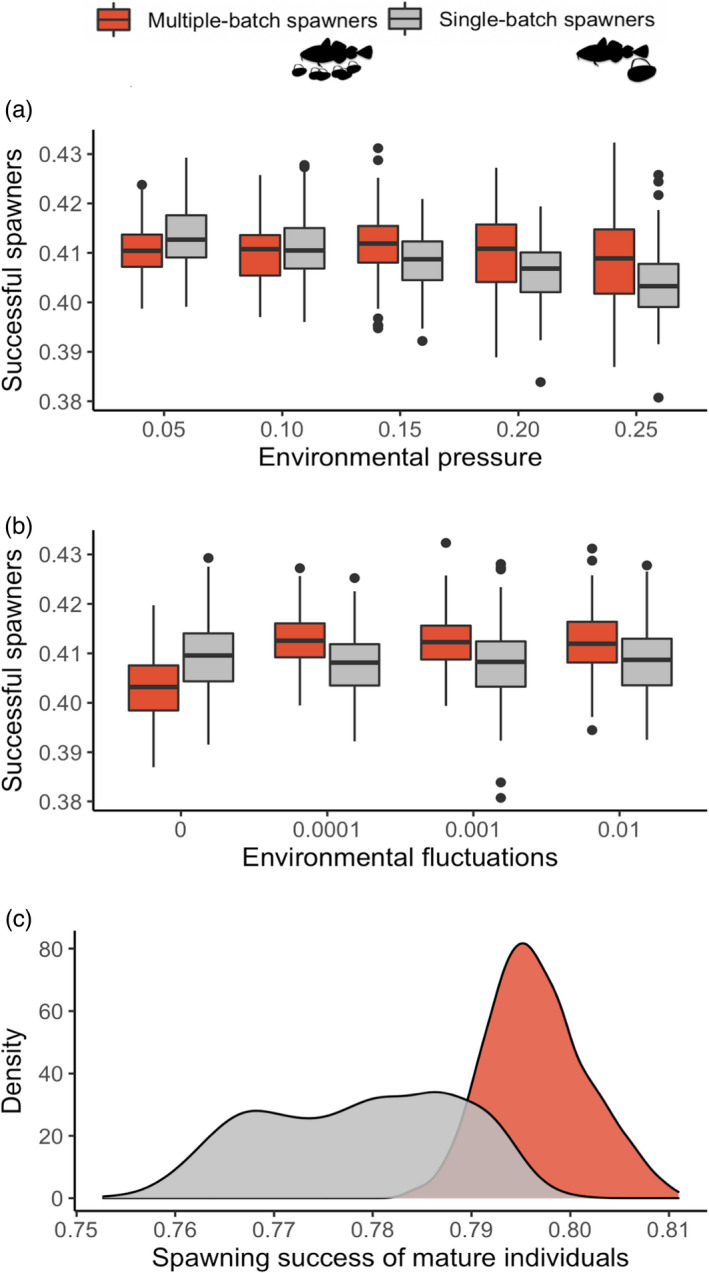
The effect of multiple‐batch spawning strategy on spawning success under the components of increasing environmental stochasticity. The two boxplots show the difference in the proportion of successful spawners in a population of multiple‐batch (red) and single‐batch spawners (grey) against increasing environmental pressure (a) and fluctuations (b) applied to batch survival. The proportions were calculated as the total number of all successful events of all mature individuals in the last 300 years and divided by the total sum of succeeded and failed spawning events of all mature individuals in the last 300 years. The density plot (c) illustrates concentrated values over the interval of successful spawning seasons of mature individuals, being right‐skewed and more concentrated in the presence of a multiple‐batch spawning strategy

The proportion of successful spawning seasons in a population, when an individual produced at least one successfully surviving offspring, reaching age 3 (recruitment age when juveniles become catchable by fishing), per season, differed between populations of multiple‐batch and single‐batch spawners (Table S5). The average frequency of such occasions was significantly higher and more consistent in multiple‐batch spawning populations when environmental conditions were less predictable and more stressful (Figure 5a,b). In contrast, the scenarios with no environmental fluctuations (0) and low pressure applied to batch survival (0.05) were more favourable to single‐batch spawning populations which exhibited higher spawning success under such conditions (Figure 5a,b). The success probability of mature fish was on average higher and more predictable in the presence of the multiple‐batch spawning strategy (Figure 5c).

## 4 DISCUSSION

It has been hypothesized that multiple‐batch spawning in fishes might comprise a bet‐hedging strategy and yield high fitness returns (e.g. Hutchings & Rangeley, 2011). In the present study, we used Atlantic cod as a model species and extended the eco‐evolutionary mechanistic model of Kuparinen et al. (2012) to theoretically and empirically explore the hypothesis by evaluating the fitness consequences of such a risk‐spreading trait.

The most interesting finding to emerge from the simulations of our empirically parameterized eco‐evolutionary model is that the costly multiple‐batch spawning strategy can constitute a bet‐hedging trait under sufficiently uncertain natural environments. The multiple‐batch spawning strategy of individuals exposed to fluctuating environmental conditions served to reduce the variance in arithmetic mean fitness across generations, reflecting the decreasing variance in offspring output within generations.

The fitness of spawners under stochastic environments is governed by the geometric mean in their reproductive success (Gillespie, 1974) rather than the average mean, which fails to account for environmental variability (Lewontin & Cohen, 1969). We followed the across‐generational fitness and found that the multiple‐batch spawning strategy maximizes geometric mean fitness by lowering the across‐generational variance in arithmetic mean fitness (Figure 3). These fitness dynamics are indicative of a bet‐hedging strategy. Once the arithmetic mean fitness decreases due to the appended costs of the strategy, and when the decrease in arithmetic mean is synchronous with the increase in across‐generational geometric mean fitness, multiple‐batch spawning performs as a bet‐hedging (Figure 4) and evolutionarily outperforms the costless single‐batch spawning strategists (Figure 3 grey‐shaded area).

A fundamental component of our results lies in the qualitative, rather than quantitative, output. Firstly, we are primarily interested in the directions in which multiple‐batch spawning confers advantages, such as reduced long‐term variance in fitness or increased probability of non‐zero reproductive spawning years, rather than numerical differences between multiple‐batch spawners and single‐batch spawners. Secondly, although one might be tempted to conclude that our statistically significant quantitative differences are of little biological consequence, we would caution against such a conclusion. Seemingly small phenotypic differences at the species or population level can have considerably greater consequences within a multi‐species or ecosystem context (Bassar et al., 2010; El‐Sabaawi et al., 2015).

By producing several batches per spawning season (Kjesbu et al., 1996; Roney et al., 2018), batch spawners distribute eggs among multiple batches, thereby spreading the risk on both spatial and temporal scales. This results, on average, in increased survival probabilities for each egg and, as identified in the present study, a decreased realized populational mortality rate, highlighting how early life stages shape the vital rates of adults (Hjort, 1914). Concomitantly, the high spawning success and lower reproductive variance associated with multiple‐batch strategy under highly unpredictable and intense environmental perturbations presented the underlying reason for the increased fitness that was maximized across generations.

On the other hand, under scenarios in which temporal and spatial fluctuations were absent and all batches endured equal environmental pressure, each time that a fish spawned, the average output of successfully surviving offspring varied considerably within and across generations, resulting in a higher arithmetic and lower geometric mean fitness compared to the single‐batch spawning strategy. This suggests that, apart from bypassing the physiological constraints imposed by egg shedding, solely from the isolated perspective of egg batch effects on fitness dynamics, the costs of the multiple‐batch spawning strategy are too high and fitness benefits too low to pay off under fairly stable environmental circumstances in the long run. Therefore, multiple‐batch spawning of Atlantic cod is plausibly selected for to endure greater natural stochasticity. The outcome could be common among batch spawning fish species that are exposed to similar selective pressures and that share similar trade‐off in life‐history traits and costs of reproduction (Longhurst, 2002).

Several studies have demonstrated why, in a purely spatially varying or a fine‐grained environment (Levins, 1968), a bet‐hedging response would not evolve or be favoured (Haaland et al., 2020; Moran, 1992). A costly strategy requires a certain ratio of spatial‐temporal variation and a trade‐off in fitness to be advantageous and adaptive. Environmental settings where individuals face different environmental variability only on a spatial scale within generations gain fitness additively as reflected by the arithmetic mean fitness, whereas environmental variability on an exclusively temporal scale across generations gain fitness multiplicatively as reflected by the geometric mean fitness (Haaland et al., 2020).

The externally applied environmental stochasticity in our model might have, to some degree, generated such a fine‐grained environmental setting as it was newly drawn for each batch of every female within each generation. However, there are at least three reasons why our simulations also accounted for temporal fluctuations, thus steering the environments towards coarser graininess. Firstly, the environmental setting of fluctuating scenarios (Figure 1) was newly drawn for every spawning season, adding the temporal aspect of changes from one year to the next and, as such, generated conditions where a bet‐hedging strategy could unfold. Secondly, the presence of temporal variability was additionally endorsed by our calculations of arithmetic and geometric fitness, for which the values were never equal, indicating that the environments were not constant but rather fluctuating through time because of external stochasticity simulating the biotic and abiotic change (Orr, 2009). And lastly, the 20 diverse but discrete environmental scenarios enabled us to obtain a broad glimpse into a coarse‐grained setting. Notwithstanding these points, the modelling of a more detailed year‐to‐year interchangeable environmental pressure on spawning dynamics might reveal further effects of the multiple‐batch spawning strategy on fitness components of cod populations.

The mechanistic model that we used is, as any other model, a simplification of a natural system, one that includes simplifying assumptions; these need to be considered for model interpretation. For instance, one of the assumptions was a uniform distribution of egg sizes and abundance within and among egg batches. This simplification could reduce the effect of the selective pressure on larger phenotypes. Kjesbu (1989), Kjesbu et al. (1996), for example, found that abundance and mean weight of eggs of Norwegian coastal cod varied among batches and spawning seasons. While the egg abundance follows a dome‐shaped curve, which can be right or left‐skewed, the mean size of offspring decreases towards the end of the spawning period (Kjesbu et al., 1996; Roney et al., 2018). In addition, it is known that natural selection can favour variability in egg size within batches (Koops et al., 2003; Olofsson et al., 2009). Our primary reason for omitting these relationships was to isolate the multiple‐batch production trait and to focus solely on its unique diversifying influence on fitness.

Taking into account the model's assumptions, we can conclude that multiple‐batch spawning contributes to the long‐term persistence of cod genotypes under stochastic environmental conditions, where the cost–benefit ratio is low, and reduces the probability of reproductive failure (Figure 5). In contrast, the strategy of laying all eggs at once in every spawning season would increase the survival uncertainty of the batches, resulting in a game with two outcomes: success or complete reproductive failure. Here, we found that the within‐ and across‐generational reproductive variance associated with such a single‐batch spawning strategy would be higher and result in lower fitness when faced with strong environmental stochasticity, as it is more sensitive to the number of complete reproductive failures.

The novelty of our work lies in the direct comparison of across‐generational geometric mean fitness between risk‐spreading and nonrisk‐spreading cod populations. Our modelling approach provided empirically anchored support for the hypothesis that multiple‐batch spawning is advantageous and adaptive in stochastically variable environments. Following Simons (2011), such a direct test falls at least into the second highest strength of evidence category (V), which involves the bet‐hedging trait to be validated whether it is favoured under relevant varying environments. We manipulated the magnitude of selection through the degree of environmental stochasticity and found that the presence of the trait significantly stabilized fitness of cod under most fluctuating environmental settings.

Extensive theoretical work has been developed on the maximization of geometric fitness under stochastic conditions (Cohen, 1966; Lewontin & Cohen, 1969; Simons, 2002; Starrfelt & Kokko, 2012; Yoshimura & Clark, 1991), but some ambiguities persist (Metz et al., 1992; Sæther & Engen, 2015). For example, Tal and Tran (2020) have stressed the need to re‐consider or upgrade the approach of the maximized geometric mean fitness in the search of a bet‐hedging trait. In the present study, we derived the mean geometric fitness using standard nth roots of the multiplicative approach (Seger & Brockmann, 1987). We observed the cod population in our model to be flexible; whenever the rate of egg batch mortality equalled or exceeded 0.30, the population collapsed within the first 100 years and became extinct. This makes ecological sense, given that several mortality rates were combined in our model during the lifespan of an individual to simulate natural environmental conditions, including increased juvenile mortalities (Anderson & Gregory, 2000), and survival and reproduction costs (Lambert & Dutil, 2000). That said, a more detailed exploration of extinction probabilities was beyond the scope of our study, and we did not pursue the differences between the two genotype populations in their resilience to extinction.

Another ambiguity involves false dichotomy. Starrfelt and Kokko (2012) concluded that the usual partitioning of conservative and diversification bet‐hedging should not be treated only as two discretely separate categories. Their point was that the benefit of bet‐hedging can also derive from their combination; reduced variance on the individual fitness level can represent a conservative part of the trait, and reduced fitness correlations among individuals can present a diversifying part of the trait. As a result, they proposed that the two strategies be considered as endpoints along a continuous scale. Recent theoretical research illustrated how they might coexist (Haaland et al., 2019). Similarly, the trait examined in the present study seems to encompass elements of both conservative and diversification bet‐hedging. Multiple‐batch spawning is a positive size‐based trait that reduces individual‐level variance in fitness and, thus, resembles a conservative type of bet‐hedging (Haaland et al., 2020; Starrfelt & Kokko, 2012), while at least two rationales could reflect the diversifying characteristics of bet‐hedging: the stock demographic structure and subpopulation connectivity (e.g. *stock complex*).

The benefits of a multiple‐batch spawning might be integrated across multiple levels of biological organization. For instance, Shelton et al. (2015) showed that spawning stock age structure has a significant effect on the recruitment dynamics of Atlantic cod. Therefore, the eradication of one cohort due to a natural catastrophe or anthropogenic impact such as overfishing (Hutchings, 2005) could reduce the diversity in size‐structure of a population and consequently increase the fitness correlation among individuals or, in another words, repress the diversifying bet‐hedging fitness benefits that the multiple‐batch spawning of unfished populations might offer.

A spatial distribution in metapopulation structure could also be detrimental through larval dispersal or even adult migration (Hu & Wroblewski, 2009). Genetic analyses of North Sea and coastal inshore and offshore cod populations in the Norwegian Skagerrak have revealed an alternating connectivity among coexisting subpopulations (Knutsen et al., 2004), which display a fine‐scaled differentiation in life‐history characteristics (Kuparinen et al., 2016). Although the subpopulations appear to be more linked in some years than others (Knutsen et al., 2004), the connectivity ensures higher diversity of life histories in a subpopulation and minimizes the correlation in spawning potential of stock. In contrast, populational fragmentation via local extinctions (Hutchings & Myers, 1994) could disrupt the connectivity with neighbouring areas through perished adult links or decreased recruit inflow, which can have a far‐reaching effect in maintaining gene flow to locally depleted stocks or in mitigating poor spawning seasons (Stenseth et al., 2006). Thus, the benefits of dividing eggs into several batches could, hypothetically, arise from both individual‐ and population‐level considerations. However, to test our speculations, we would need to further explore the fitness correlations among individuals of each strategists.

Our results highlight that producing several egg batches per spawning period increases spawning success of batch spawners. This might be related to prolonged spawning seasons expanding the time window for optimal abiotic conditions to occur and influence the variation in survival probability within an egg batch. Early‐stage survival of fish is known to be a highly stochastic process (Ohlberger et al., 2014), and multiple factors contribute to successful recruitment and fitness, from favourable abiotic conditions driven by currents (Hjort, 1914) to biotic processes of bottom‐up control (Cushing, 1990), habitat complexity (Theodorou et al., 2013), and density‐dependent regulation (Fromentin et al., 2001; Kuparinen et al., 2014). For example, given that the probability of egg and larval transport to suitable habitats can be strongly influenced by the timing of release coinciding with the favourable environmental conditions (e.g. Huserbråten et al., 2018), it is conceivable that the multiple‐batch spawning strategy would increase the probability of these coincidentally occurring events. Multiple‐batch spawning is also likely to affect the dynamics of a temporal ‘match’/‘mismatch’ between the peak abundance of larvae and their prey, such that variability in offspring production is inversely related to the length of spawning season (Mertz & Myers, 1994). The empirical modelling study of cod by Kristiansen et al. (2011) supports our findings; offspring survival increased as the spatial and temporal overlap between fish larvae and their prey increased. The duration of overlap during the spawning period was more beneficial to recruitment than the co‐occurrence of peak prey and larvae abundance, which further underscores the risk‐spreading benefits of prolonged spawning among batch spawners.

In summary, we show that the risk‐spreading mechanism of producing multiple batches facilitates cod to endure greater environmental stochasticity. The number of batches, which increases with maternal body size, contributes significantly to across‐generational fitness of populations experiencing highly unpredictable environmental perturbation. This relation invokes new rather applicative questions such as could a size‐selective fishing pressure have a magnified impact on the effective size‐structure of a cod population and cod‐like species because of a size‐related spawning trait? Could the fishing of larger individuals abduct the population of the security mechanism that the bet‐hedging ensure and consequently diminish its portfolio effect? Since several stocks of batch spawning fish species have been subjected to overfishing and are still in a rebuilding phase (e.g. Hutchings & Kuparinen, 2020), the role of traits that enable populations to reduce susceptibility to environmental variation may be vital to species recovery and success. Bet‐hedging strategies such as batch spawning can yield high fitness returns and should therefore be integrated in proactive stock management, including the setting of reference points. Spawning type influences the reproductive dynamics of stocks, which is the underlying reference for management implications. We suggest that recognizing stress‐coping mechanisms of species and understanding their dynamics under naturally and anthropogenically induced stressful conditions is a critical issue that needs to be tackled to fill the knowledge gaps on fitness dynamics of harvested stocks and to achieve sustainable use of natural resources.

## CONFLICT OF INTEREST

We have no conflicts of interest to disclose.

## Supporting information

Supplementary Material

## Data Availability

Data for this study are available at the Dryad Digital Repository: https://doi.org/10.5061/dryad.g1jwstqn0.
