# Supplementary material for: Multiple‐batch spawning as a bet‐hedging strategy in highly stochastic environments: An exploratory analysis of Atlantic cod
Source: Evol Appl. 2021 Jun 10;14(8):1980–92. doi: 10.1111/eva.13251 (PMC8372085; doi:10.1111/eva.13251)
Supplement: Supplementary file 1 — Supplementary Material [file EVA-14-1980-s001.docx]

**Supplementary material to:**

Multiple-batch spawning as a bet-hedging strategy in highly stochastic environments: an exploratory analysis of Atlantic cod

Contents: Supplementary Table S1, S2, S3, S4, S5, S6 and Fig. S1, S2, S3**Table S1.** Combinations of simulated runs for each of four scenarios of multiple-batch and single-batch spawning Atlantic cod population in a reproductive environmental stochastic and non-stochastic scenario, showing the values of pre-set parameters.

|  | Non Stochastic environment | Stochastic environment |
| --- | --- | --- |
| Multiple-batch spawners | Environmental pressure  {0.05, 0.10, 0.15, 0.20, 0.25}  x  Environmental fluctuations  {0} | Environmental pressure  {0.05, 0.10, 0.15, 0.20, 0.25}  x  Environmental fluctuations  {0.0001, 0.001, 0.01} |
| Single-batch spawners | Environmental pressure  {0.05, 0.10, 0.15, 0.20, 0.25}  x  Environmental fluctuations  {0} | Environmental pressure  {0.05, 0.10, 0.15, 0.20, 0.25}  x  Environmental fluctuations  {0.0001, 0.001, 0.01} |

**Table S2.** Values of alpha and beta shape parameters used to produce beta distribution in the simulations for each corresponding scenario with pre-set mean and variance values in batch mortality rate.

| **Mean** | **Variance** | **CV** | **α** | **β** |
| --- | --- | --- | --- | --- |
| 0.05  0.05  0.05  0.10  0.10  0.10  0.15  0.15  0.15  0.20  0.20  0.20  0.25  0.25  0.25 | 0.01  0.001  0.0001  0.01  0.001  0.0001  0.01  0.001  0.0001  0.01  0.001  0.0001  0.01  0.001  0.0001 | 2.00  0.63  0.20  1.00  0.32  0.10  0.67  0.21  0.07  0.50  0.16  0.05  0.40  0.13  0.04 | 0.1875  2.3250  23.7000  0.8  8.9  89.9  1.7625  18.9750  191.1000  3.0  31.8  319.8  4.4375  46.6250  468.5000 | 3.5625  44.175  450.3000  7.2  80.1  809.1  9.9875  107.5250  1082.9000  12.0  127.2  1279.2  13.3125  139.8750  1405.5000 |

**Table S3.** Differences in population size, biomass, variance in within-generational reproductive output and realised mortality rate between the multiple-batch and single-batch spawning Atlantic cod populations over all simulated environmentally stochastic scenarios. The data have been explored with Welch two sample t-test summary statistics.

|  | Source of variation | t | df | Multiple-batch spawner | | Single-batch spawner | | | CI95% | p |
| --- | --- | --- | --- | --- | --- | --- | --- | --- | --- | --- |
| Population size | Spawning type | 9.4824 | 1990.3 | | 5440.167 | | 5181.634 | [205.06, 312.00] | | < 2.2e-16* |
| CV in population size | Spawning type | -9.4473 | 1984 | | 2.631 | | 2.812 | [-0.2187, -0.1435] | | < 2.2e-16* |
| Population biomass | Spawning type | 14.317 | 1997.4 | | 4605.859 | | 4518.849 | [75.0906, 98.9285] | | < 2.2e-16* |
| CV in population biomass | Spawning type | -14.143 | 1873.5 | | 1.300 | | 1.661 | [-0.4114, -0.3112] | | < 2.2e-16* |
| Within-generation $\sigma_{{RO}_{G}}^{2}$ | Spawning type | -2.888 | 1991.4 | | 18.1375 | | 18.2348 | [ -0.1635, -0.0312] | | 0.0039* |
| Realised mortality rate | Spawning type | -25.739 | 1622.3 | | 0.170 | | 0.171 | [-0.0010, -0.0009] | | < 2.2e-16* |

**p* < .05

**Table S4**. The effect of applied environmental fluctuations and pressure to batch survival on population size, biomass, variance in within-generational reproductive output and realised mortality rate of multiple-batch and single-batch spawning cod populations. The data has been analysed with a non-parametric Kruskal-Wallis test. Post-hoc pairwise Dunn’s test of multiple comparisons, performed after each Kruskal-Wallis test, revealed the nonstochastic scenario (where no environmental fluctuations were added to batches) to be the only reason for significant difference between pairs. Contrary, the significant difference among rates of environmental pressure had a pairwise distinction.

|  | Source of variation |  | **Multiple-batch spawner** | | **Single-batch spawner** | |
| --- | --- | --- | --- | --- | --- | --- |
|  |  | *df* | *X^2^* | *p* | *X^2^* | *p* |
| Population size | Environmental pressure | 4 | 394.25 | < 2.2e-16 * | 959.04 | < 2.2e-16 * |
|  | Environmental fluctuations | 3 | 532.01 | < 2.2e-16 * | 0.19708 | 0.9781 |
| CV in population size | Environmental pressure | 4 | 648.25 | < 2.2e-16 * | 812.33 | < 2.2e-16 * |
|  | Environmental fluctuations | 3 | 2.3581 | 0.5015 | 2.449 | 0.4846 |
| Population biomass | Environmental pressure | 4 | 250.8 | < 2.2e-16 * | 958.61 | < 2.2e-16 * |
|  | Environmental fluctuations | 3 | 561.96 | < 2.2e-16 * | 0.18518 | 0.9799 |
| CV in population biomass | Environmental pressure | 4 | 325.45 | < 2.2e-16 * | 952.5 | < 2.2e-16 * |
|  | Environmental fluctuations | 3 | 536.01 | < 2.2e-16 * | 0.18668 | 0.9797 |
| Within-generation $\sigma_{{RO}_{G}}^{2}$ | Environmental pressure | 4 | 12.53 | 0.0138* | 69.572 | 2.8e-14* |
|  | Environmental fluctuations | 3 | 161.04 | < 2.2e-16 * | 5.0138 | 0.1708 |
| Realised mortality rate | Environmental pressure | 4 | 36.285 | 2.53e-07* | 42.661 | 1.22e-08* |
|  | Environmental fluctuations | 3 | 462.69 | < 2.2e-16 * | 5.7393 | 0.125 |

**p* < .05

**Table S5.** Differences in fitness dynamics between the multiple-batch and single-batch spawning Atlantic cod populations over all simulated environmentally stochastic scenarios. The data have been explored with Welch two sample t-test summary statistics.

|  | Source of variation | t | df | Multiple-batch spawner | | Single-batch spawner | | CI95% | p |
| --- | --- | --- | --- | --- | --- | --- | --- | --- | --- |
| Geometric $\bar{W}_{GM}$ | Spawning type | -2.4035 | 1994.7 | | 2.0721 | 2.0742 | [ -0.0037, -0.0004] | | 0.0163* |
| Arithmetic $\bar{W}_{AM}$ | Spawning type | 3.0536 | 1980.5 | | 2.5713 | 2.5663 | [ 0.0018, 0.0082] | | 0.0023* |
| Variance in $\bar{W}_{AM}$ | Spawning type | 3.0851 | 1998 | | 8.1892 | 8.0213 | [ 0.0611, 0.2745] | | 0.0021* |
| Spawning success | Spawning type | 5.1206 | 1997.9 | | 0.4100 | 0.4084 | [ 0.0010, 0.0022] | | 3.3e-07* |

**p* < .05

**Table S6**. Environmental fluctuations and environmental pressure effects on fitness dynamics of multiple-batch and single-batch spawning cod populations. The data have been analysed with a non-parametric Kruskal-Wallis test.

|  | Source of variation |  | **Multiple-batch spawner** | | **Single-batch spawner** | |
| --- | --- | --- | --- | --- | --- | --- |
|  |  | *df* | *X^2^* | *p* | *X^2^* | *p* |
| Geometric $\bar{W}_{GM}$ | Environmental fluctuations | 3 | 8.21 | 0.0418* | 1.9806 | 0.5764 |
|  | Environmental pressure | 4 | 9.51 | 0.0496* | 6.1032 | 0.1916 |
| Arithmetic $\bar{W}_{AM}$ | Environmental fluctuations | 3 | 21.48 | 8.3e-05* | 1.5419 | 0.6726 |
|  | Environmental pressure | 4 | 2.42 | 0.6591 | 5.2314 | 0.2644 |
| Variance in $\bar{W}_{AM}$ | Environmental fluctuations | 3 | 20.30 | 0.0001* | 0.92697 | 0.8189 |
|  | Environmental pressure | 4 | 3.30 | 0.5084 | 7.7291 | 0.1020 |
| Spawning success | Environmental fluctuations | 3 | 280.81 | < 2.2e-16 * | 6.5658 | 0.0871 |
|  | Environmental pressure | 4 | 16.74 | 0.0022* | 232.53 | < 2.2e-16 * |

**p* < .05

**
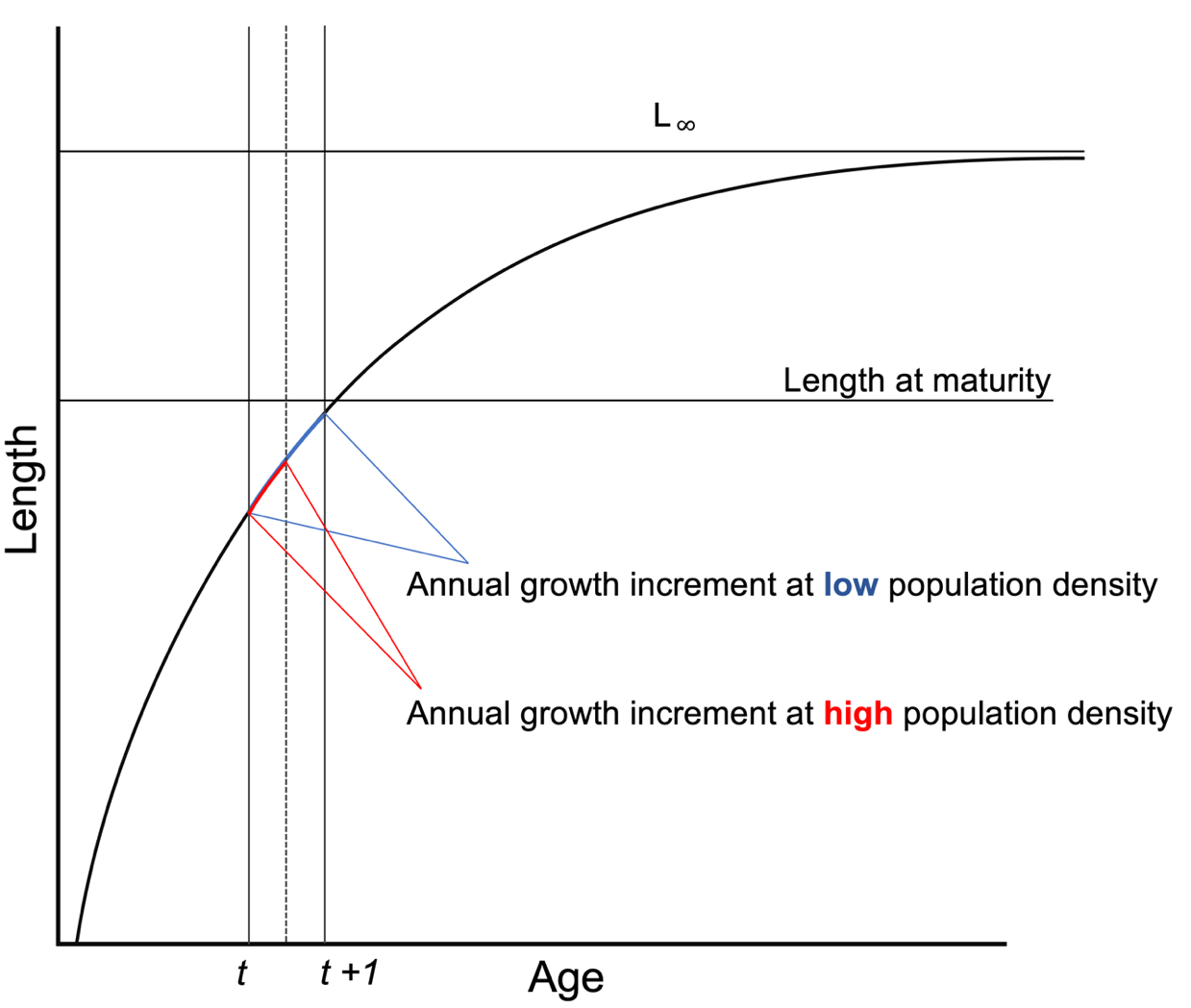
**

**Fig. S1.** Individual’s growth increment. Schematic figure from Kuparinen et al. 2012 illustrates the annual growth along the von Bertalanffy growth curve. The growth of an individual followed the growth trajectory at low populational density (blue) and was reduced at high populational density (red) if the population reached its carrying capacity.


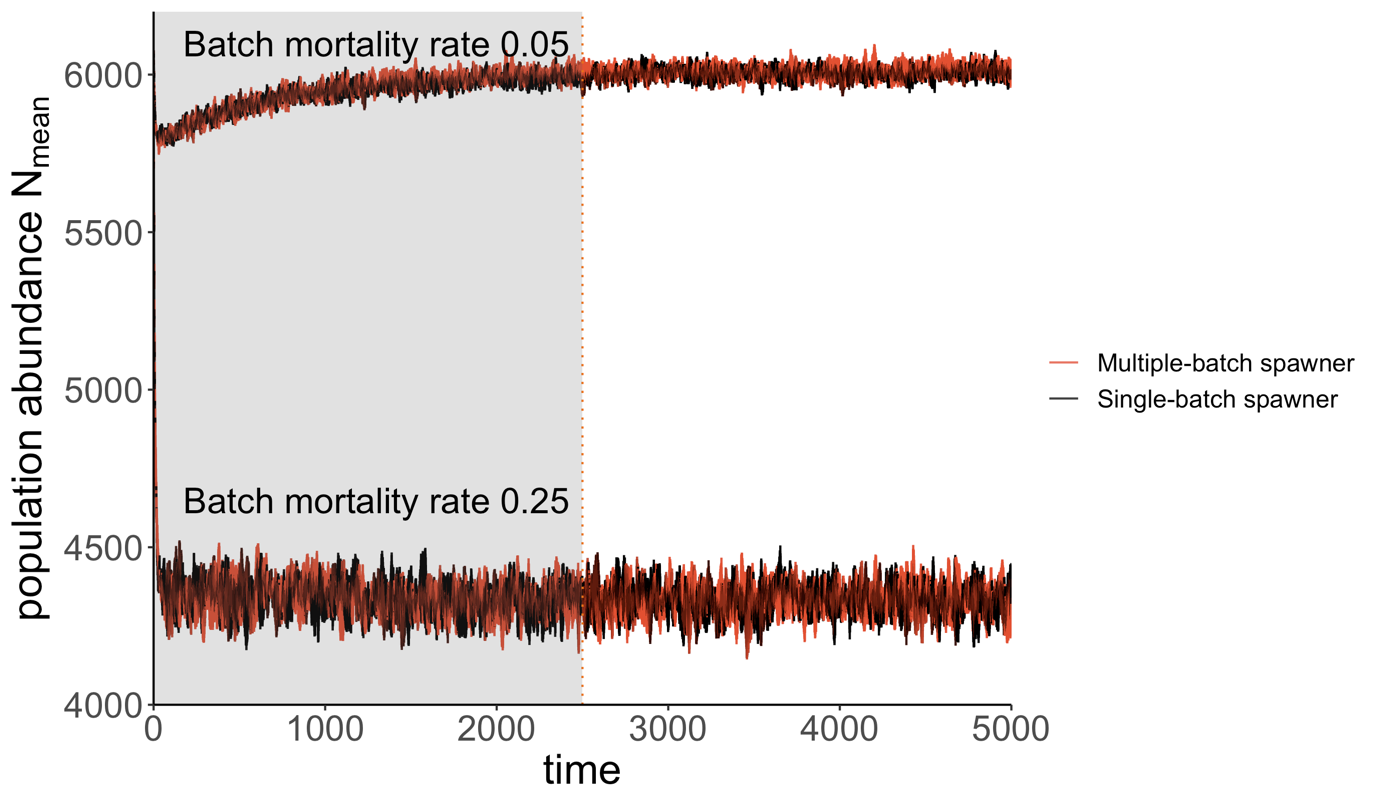
**Fig. S2.** Dynamics in abundance of the cod population with multiple-batch spawning strategy (red) and single-batch spawning strategy (black), under the highest and the lowest applied batch mortality rates; 0.25 and 0.05, respectively. The grey shaded area indicates the part of the simulation that has been disregarded for the analysis of life-history traits.

**
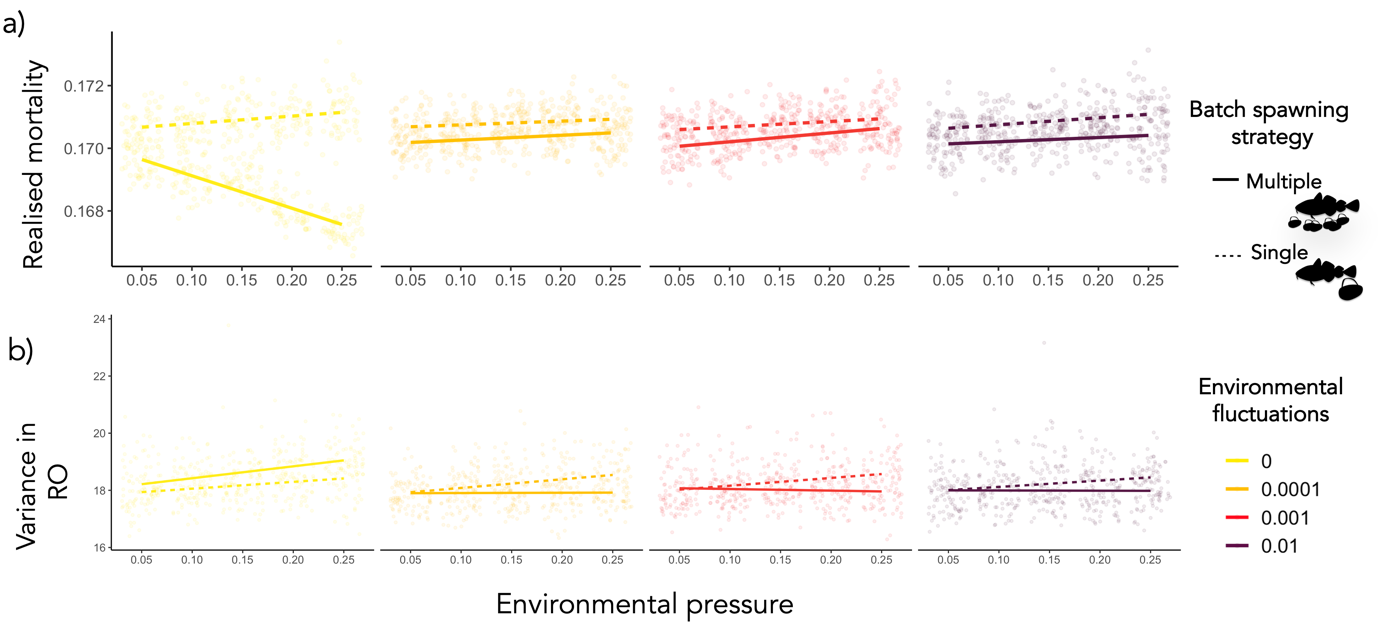
Fig. S3.** Realised populational mortality rate (a) and within-generational variance in reproductive output (b) plotted against environmental pressure applied to batch surviaval (x-axis) and grouped by increasing environmental fluctuations (colours). Data and linear regression trends are illustrated by a solid line for multiple-batch spawners and dashed line for a single-batch spawners. Each data point represents the mean value in observed variable of all matured individuals tracked in 300 years per every run.
